# Amino acid starvation and iron limitation facilitate the biofilm formation of *Klebsiella pneumoniae* within urine

**DOI:** 10.1016/j.bioflm.2026.100347

**Published:** 2026-01-16

**Authors:** Xinming Pan, Yinchu Zhu, Yan Zhang, Jie Zhao, Xing Gao, Caiying Li, Yong Yu, Jiale Ma

**Affiliations:** a MOE Joint International Research Laboratory of Animal Health and Food Safety, College of Veterinary Medicine, Nanjing Agricultural University, Nanjing, 210095, China; b Key Lab of Animal Bacteriology, Ministry of Agriculture, Nanjing, 210095, China; c WOAH Reference Lab for Swine Streptococcosis, Bacterial Pathogenesis Research Group, Nanjing, 210095, China; d Institute of Animal Husbandry and Veterinary Sciences, Zhejiang Academy of Agricultural Sciences, Hangzhou, 310021, China; e Shihu Scenic Area Administration of Suzhou City(Suzhou South China Tiger Breeding Base), Suzhou, 215168, China; f Nanjing Dr. Vet Health Management Co., Ltd., Nanjing, 210095, China; g Jiangsu Key Laboratory of Zoonosis, Yangzhou University, Yangzhou, 225009, China

**Keywords:** *Klebsiella pneumoniae*, Biofilm, Urine, c-di-GMP, Exopolysaccharides

## Abstract

Biofilm formation is a critical virulence mechanism in pathogens such as *Klebsiella pneumoniae*, a Gram-negative, encapsulated bacterium that has emerged as a zoonotic threat capable of infecting both humans and animals. Its biofilm-forming ability is closely associated with catheter-related and urinary tract infections. Given its potential to cross species barriers and cause significant public health concern, elucidating the environmental cues and conserved molecular pathways driving biofilm formation is essential for developing cross-species prevention strategies. Here we found that *K. pneumoniae* exhibited significantly greater biofilm-forming efficiency in urine than in nutrient-rich medium under comparable biomass conditions. Transposon-insertion sequencing (Tn-seq) identified 19 fitness genes essential for optimal growth in urine, most involved in the *de novo* biosynthesis of amino acids, particularly arginine, methionine, and isoleucine. Urine represents an amino acid-starved (AAS) environment for *K. pneumoniae*, modulating c-di-GMP signaling to promote biofilm formation. Eight diguanylate cyclase (DGC, c-di-GMP synthesis) genes, four phosphodiesterase (PDE, c-di-GMP degradation) genes, and four DGC + PDE genes were significantly regulated in response to urine. Furthermore, transcriptomic analysis comparing *K. pneumoniae* grown in urine with that grown in M9 medium revealed significant activation of genes associated with exopolysaccharide (EPS) biosynthesis, including those encoding lipopolysaccharides (LPS), capsules, peptidoglycan, and enterobacterial common antigen (ECA). Notably, *K. pneumoniae* increases EPS biosynthesis under the iron-limited conditions in urine, further promoting biofilm development. In conclusion, AAS-mediated c-di-GMP signaling and iron limitation are key drivers of biofilm formation by *K. pneumoniae* in urine, providing mechanistic insights that may guide strategies to disrupt biofilm formation.

## Highlights

• *K. pneumoniae* exhibits greater biofilm-forming efficiency in urine than medium.
• Amino acid starvation in urine modulates c-di-GMP to promote biofilm formation.
• Urine triggers genes for exopolysaccharide biosynthesis, enhancing biofilm matrix.
• Iron limitation in urine enhances EPS gene expression and biofilm formation.

## 1 Introduction

Biofilm formation is a major virulence mechanism for many bacteria, contributing to persistent infections by shielding cells within a protective extracellular matrix. In fact, a significant portion of bacterial infections—often cited as up to four-fifths—are thought to involve biofilms. *Klebsiella pneumoniae*, a Gram-negative encapsulated bacterium, is notorious for causing hospital-acquired pneumonia, urinary tract infections, bloodstream infections, and other severe conditions [1,2]. Its ability to form robust biofilms on medical devices, including catheters, implants, and ventilator components, underlies its tenacity in clinical settings. In urinary tract infections, the shift from free-swimming to surface-attached states enables *K. pneumoniae* to ascend the urethra and establish bladder colonization, exacerbating its pathogenicity. Notably, previous studies have shown that *K. pneumoniae* forms its most abundant biofilms in natural human urine, further highlighting the urinary tract as a highly conducive environment for its persistence [3].

Studies characterizing the biofilm architecture of *K. pneumoniae* indicate that it is mainly constituted by extracellular polymeric substances [4], with lipopolysaccharides and capsular polysaccharides serving as the principal structural elements [5,6]. Capsular polysaccharides are indispensable for the establishment of biofilms, but they may also reduce surface attachment by shielding fimbrial adhesins [7,8]. Interestingly, the absence of capsule exerts contrasting effects depending on the strain background: in hypermucoviscous K1 isolates it facilitates biofilm formation, whereas in K124 isolates it diminishes it, suggesting that capsule levels together with adhesion systems jointly determine biofilm development [9]. Adhesion to surfaces is largely governed by fimbrial organelles encoded by chaperone–usher operons, with type I fimbriae (*fimA*–*K* cluster) and type III fimbriae (*mrkA–I* cluster) as the most relevant representatives [10,11]. Among them, type III fimbriae are consistently correlated with enhanced biofilm formation [12], while the influence of type I fimbriae is less predictable and can vary with the host niche or experimental setting [13,14]. A broad set of genes has been implicated in the regulation of biofilm production in clinical isolates, such as those involved in allantoin metabolism (*allS*), capsular polysaccharide biosynthesis (*treC*, *cpsD*, *wzc*, *wabG*, *wcaG*, *rmpA/A2*, *wzyk2*, *magA*), siderophore generation (*iutA*), polysaccharide and adhesin production (*pgaA*, *pgaB*, *pgaC*, *bcsA*), fimbrial components (*fimA*, *fimH*, *mrkD*, *mrkA*), quorum-sensing signaling (*luxS*), and colanic acid synthesis [7,15,16].

After transitioning from the gut or external sources, *K. pneumoniae* encounters the urinary tract, an environment characterized by limited nutrients. *Ex vivo* growth in human urine provides a simplified model to mimic some of these urinary conditions. To overcome iron restriction during urinary tract infections (UTIs), the bacterium employs a variety of iron acquisition systems, reflecting a high level of redundancy [17]. As in many bacteria, the transcriptional regulator Fur coordinates iron homeostasis in *K. pneumoniae* and influences multiple virulence traits, including the ability to form biofilms [18]. Specifically, Fur modulates the transcription of type I [18,19] and type III [20] fimbrial genes, as well as associated fimbrial regulators, in response to iron availability. The urinary tract is also a nutrient-limited environment, particularly deficient in nucleotides and certain amino acids. Prior research has demonstrated that *de novo* synthesis of nucleotides, arginine, and methionine is essential for uropathogenic *E. coli* (UPEC) colonization [21]. However, whether these biosynthetic pathways contribute to biofilm formation in *K. pneumoniae* remains to be determined.

Bacterial proliferation in urine is challenged by amino acid starvation (AAS) [22,23], a condition representing extreme limitation in bacterial amino acid metabolism, including both insufficient nitrogen intake due to defective amino acid synthesis and inadequate uptake or biosynthesis of essential amino acids. Under such conditions, bacteria produce various signaling molecules, including ppGpp, sigma factors, and cyclic diguanylate monophosphate (c-di-GMP), which coordinate growth, metabolic activity, and virulence [24,25]. *Salmonella Typhimurium* exhibits a rise in c-di-GMP levels upon exposure to l-arginine during infection [26]. c-di-GMP serves as a pivotal intracellular signal that modulates bacterial movement and surface attachment, facilitating the establishment of biofilms [27,28]. The synthesis of this molecule is catalyzed by diguanylate cyclases possessing GGDEF domains, while its degradation is facilitated by phosphodiesterases containing EAL domains [29]. DGCs and PDEs dynamically adjust c-di-GMP levels in response to environmental cues, modulating the switch from motile to sessile lifestyles [30,31]. For instance, mutations in functional PDEs result in local accumulation of intracellular c-di-GMP, which are associated with enhanced biofilm formation and decreased motility. In *K. pneumoniae*, c-di-GMP-associated genes are widespread; for example, strain MGH 78578 harbors 33 genes encoding DGC, PDE, or both types of domains [32], including *mrkJ*, involved in type III fimbriae synthesis [33]. Proteins MrkH and CgsD carry a c-di-GMP-binding PliZ domain [34,35], and regulate type III and curli fimbriae production, respectively, to facilitate biofilm development. Additionally, BcsE possesses a recently characterized GIL c-di-GMP-binding domain and enhances biofilm formation by upregulating cellulose synthesis [36].

This research investigates how *K. pneumoniae* adapts to amino acid starvation and iron limitation in human urine, focusing on the regulatory mechanisms that modulate biofilm formation and their role in enhancing bacterial fitness during growth in urine. Numerous factors influence the establishment and persistence of biofilms in *K. pneumoniae*, emphasizing the critical role of this process in bacterial fitness and pathogenicity.

## 2 Methods

### 2.1 Bacterial strains and plasmids

All strains and plasmids employed in this work are detailed in Table S1. Construction of the transposon library and derivation of the mutant isolates were performed using *K. pneumoniae* strain Bckp021 as the genetic background for all mutagenesis procedures. All *K. pneumoniae* isolates were cultivated at 37 °C using either Luria–Bertani broth, minimal M9 medium, or filtered human urine as indicated. Cultures were incubated under gentle agitation (50 rpm) inside a sealed incubation chamber that was equilibrated to ∼2.5 % oxygen using a defined gas mixture. When required, antibiotics were supplemented at the following concentrations: ampicillin at 100 μg/mL, kanamycin at 50 μg/mL, nalidixic acid at 30 μg/mL, and chloramphenicol at 30 μg/mL. To assess the growth of mutant strains, cultures were supplemented with arginine (0.3 or 0.03 mM), methionine (0.2 or 0.02 mM), or isoleucine (0.2 or 0.02 mM). Additionally, iron-depleted medium was prepared by supplementing M9 medium with 0.25 mM of the iron chelator 2,2′-dipyridyl.

### 2.2 DNA manipulations and plasmids construction

PCR amplification, ligation into vectors, and electroporation were carried out using standard molecular biology procedures, with reference to previously described methods unless stated otherwise [37]. All enzymes required for restriction digestion and DNA modification were sourced from Thermo Fisher Scientific (Waltham, MA, USA) and handled in accordance with the supplier's protocols. Targeted gene deletion were constructed using the phage λ Red–mediated recombination system. [38]. Standard molecular cloning techniques were employed for plasmid construction, maintenance, and transformation. For genetic complementation, target genes amplified by PCR were inserted into the pGEN-pcm vector [37] via *Nde*I and *Bam*HI cloning sites. Construct integrity was confirmed by DNA sequencing, which was outsourced to a commercial sequencing service (Shanghai Sunny Biotechnology Co., Ltd., Shanghai, China).

### 2.3 Biofilm formation assays and visual observations

Biofilm formation was assessed using a 1 % crystal violet staining approach according to established protocols [39]. Cultures were grown in 2 mL EP tubes, and the resulting biofilm formation was evaluated by recording the absorbance at 595 nm (A595), with sterile broth serving as a negative control. Each experiment was repeated three times to confirm reproducibility. Crystal violet–stained biofilms were examined by Scanning optical microscopy and by scanning electron microscopy using standard preparation workflows [40,41]. After staining, plates were allowed to dry at 37 °C for 30 min prior to inspection under a light microscope at 100 × magnification. For ultrastructural imaging, biofilms grown on glass coverslips were gently rinsed three times with PBS, fixed in 2.5 % glutaraldehyde, and then observed on a Hitachi S800 scanning electron microscope (Tokyo, Japan) operating at an accelerating voltage of 5 kV.

### 2.4 Tn-seq analysis

A transposon mutant library was constructed utilizing the pUTmini-Tn5Km2 vector, following previously established methodologies [21]. Approximately 10,000 mutants were generated per batch, and by combining 10 such batches, a total of 100,000 transformants was obtained. The complete mutant collection was cultured in LB medium at 37 °C for 12 h over three consecutive passages. This pooled culture served as the inoculum (input) for subsequent urine cultures aimed at identifying transposon mutants associated with growth fitness. After growth in urine for 12 h at 37 °C, colonies from the output cultures were collected, and the resulting cell pellets were preserved at −80 °C. Shotgun genomic DNA libraries were generated from both the input inoculum and the urine-selected output populations and sequenced as paired-end reads on an Illumina HiSeq 2000 (NextOmics, Wuhan, China). Raw sequencing reads corresponding to the transposon ends (P5 end) were extracted and filtered based on split index sequences containing transposon-specific sequences. These reads were then aligned to the *K. pneumoniae* strain Bckp021 genome (CP050834.1 for the chromosome, CP050835.1 to CP050839.1 for the plasmids) using Bowtie software [42]. Following reference protocols [43], the resulting sequencing data were processed to quantify the raw abundance of sequencing reads and catalogue the distinct insertion sites for each gene. These derived metrics subsequently served as the foundation for evaluating gene-specific fitness, enabling the discrimination of essential genetic elements.

### 2.5 Extraction and quantification of c-di-GMP

Intracellular levels of c-di-GMP concentrations in *K. pneumoniae* Bckp021 were determined by liquid chromatography–tandem mass spectrometry (LC-MS/MS) according to established protocols [44]. For each condition, bacteria were cultured for 12 h at 37 °C with gentle agitation (50 rpm) inside a sealed chamber equilibrated to ∼2.5 % oxygen; media used were either M9 or urine, and specific amino acids were added where indicated. Cell pellets were collected, washed twice with sterile PBS, and extracted in 500 μL of an organic extraction buffer (0.1 % formic acid in a mixture of acetonitrile and methanol). After incubation on ice, extracts were clarified by centrifugation and the supernatants from three successive extractions were combined, dried down, and reconstituted in water prior to analysis. Quantification was performed on an AB SCIEX QTRAP 6500+ LC-MS/MS instrument [45], and measured nucleotide levels were expressed relative to the bacterial counts of the corresponding samples to provide normalized intracellular c-di-GMP values.

### 2.6 Transcriptome analysis

Bacterial cultures were initially grown overnight in 1 mL LB at 37 °C with shaking (100 rpm). Cells were harvested, subjected to three consecutive washes with sterile PBS, and the pellets resuspended in 1 mL human urine. Those suspensions were then used to seed experimental cultures by a 1:50 dilution into either fresh urine, urine supplemented with Ile, Met and Arg, or M9 medium. Cultures were incubated for 12 h at 37 °C with gentle agitation (50 rpm) inside a sealed chamber equilibrated to approximately 2.5 % oxygen. From each culture, total RNA was purified using Qiagen's RNeasy Mini spin-column workflow, incorporating a 15-min on-column DNase I digestion carried out exactly as specified by the supplier; three biological replicates were processed per condition. RNA yields were measured using a Qubit 2.0 fluorometer, after which the samples were preserved at −80 °C until analysis. Library pools were generated following previously published strategies [46] and sequencing was carried out on an Illumina HiSeq system, producing 100-base single-end reads in line with Illumina's standard workflow. Reads were aligned to the genome of strain Bckp021 using Bowtie [42], and comparative transcriptomic analysis under low-oxygen conditions was performed as in the cited protocol [46]. Statistical testing followed the Nettleton et al. [47] approach, which transforms raw *P*-values into corresponding q-values, thereby maintaining the false discovery rate below a 5 % threshold; genes with q ≤ 0.10 and an estimated fold-change ≥2 were considered significantly differentially expressed.

### 2.7 RT-qPCR

For each culture of *K. pneumoniae* Bckp021 grown under different media conditions, total RNA was first preserved with Qiagen's RNA protect reagent and subsequently purified using the RNeasy Mini spin-column system. To remove contaminating genomic DNA, the preparations were subjected to DNase I treatment (TaKaRa). First-strand cDNA synthesis was performed immediately after RNA extraction using Takara's PrimeScript reverse-transcription reagents. Quantitative PCR assays were run on an ABI Q5 platform with SYBR Green chemistry and gene-specific primers, and target transcript abundances were calculated relative to the housekeeping gene *gapA*. [48]. Relative changes in transcript abundance were computed using the comparative CT approach (expressed as 2^−ΔΔCT^). Values are reported as the mean ± SD calculated from three independently prepared RNA samples.

### 2.8 Statistical analysis

All statistical analysis were carried out using GraphPad Prism software (version 8.0). Data are presented as mean values with their corresponding standard deviations. Statistical significance between groups was evaluated using an unpaired, two-sided *t*-test, and differences were considered significant when *P* < 0.05. In figures, significance levels are indicated by asterisks as follows: ∗*P* < 0.05, ∗∗*P* < 0.01, ∗∗∗*P* < 0.001.

## 3 Results

### 3.1 Potentiated biofilm-forming efficiency of *Klebsiella pneumoniae* in urine

**Fig. 1:**
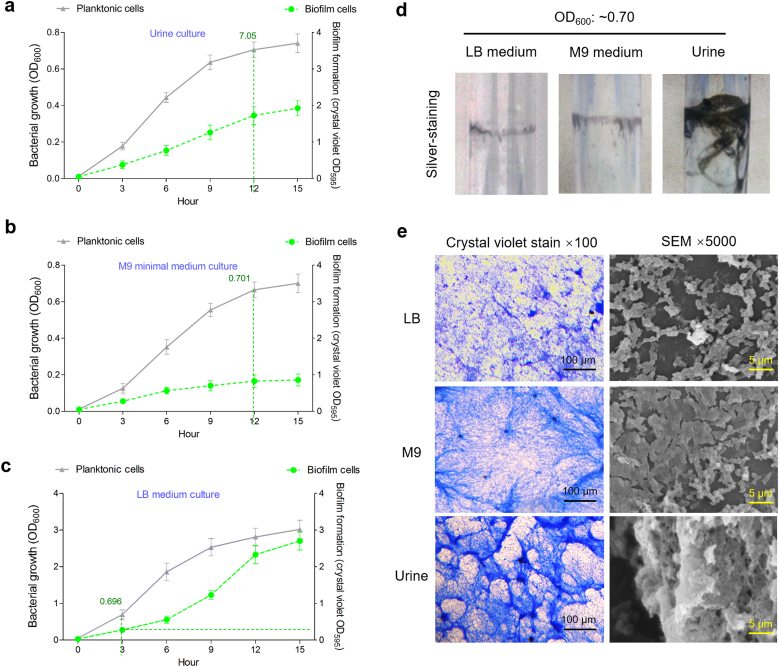
**Biofilm assays of bacterial grown in the indicated media. (a**–**c)** The growth (OD_600_) and biofilm formation (OD_595_) curves of *K. pneumoniae* cultured in urine, M9, and LB media, respectively. Data represent the mean values of three independent experiments conducted in triplicate, with error bars indicating standard deviations. (**d)** Silver-stained biofilm in the sidewalls of 2 mL EP tubes. **(e)** Biofilm images of *K. pneumoniae* cultured in urine, M9, and LB media. Crystal violet staining was employed to visualize biofilm formation, with optical microscopy conducted at 100 × magnification to assess biofilm development. SEM was utilized to examine biofilms cultivated on glass coverslips within 6-well microtiter plates, imaging at a magnification of 5000 × to observe detailed structural features. (For interpretation of the references to color in this figure legend, the reader is referred to the Web version of this article.)

Considering that typical tissue oxygen levels in the human body range between 1 % and 10 % [49], bacterial cultures were grown in 2.5 % oxygen in human urine with 50 rpm shaking to mimic *in vivo* conditions. Under these *ex vivo* conditions, the growth and biofilm formation kinetics curves of *K. pneumoniae* strain Bckp021 were detected (Fig. 1a). Similar assays were performed in M9 and LB media (Fig. 1b and c). Bacteria cultured in LB medium showed significantly higher biofilm biomass, as measured by OD_595_ values, compared to those in urine and M9 cultures. However, in the similar OD_600_ values of planktonic cells, such as 0.7, bacteria grown in urine showed the highest biofilm formation level (showed by the green curve, right Y-axis) at crystal violet OD_595_ value 1.725, followed by those in M9 and LB cultures at OD_595_ values 0.825 and 0.275, respectively. In serum-based assays using swine and rabbit sera (ethical approval for human serum was not available), *K. pneumoniae* formed significantly more biofilm than in LB or M9 media, while biofilm levels remained lower than those observed in urine (Fig. S1). We also attempted to establish a saliva-based culture model; however, the high viscosity and mucin content of saliva prevented sterile filtration unless diluted, and *K. pneumoniae* failed to grow in the filtered diluted saliva (Fig. S2). This indicated that biofilm cells constituted the highest proportion of total bacterial growth in urine, suggesting more efficient biofilm formation in this environment. Silver staining of test tubes consistently revealed strong biofilm formation by *K. pneumoniae* cultured in urine at OD600–0.7 (Fig. 1d). Microscopic analyses using crystal violet-stained slides and scanning electron microscopy (SEM) further showed the similar observations (Fig. 1e), aligning with the findings described above.

### 3.2 Urine is an environment of amino acid starvation for *K. pneumoniae* growth

**Fig. 2:**
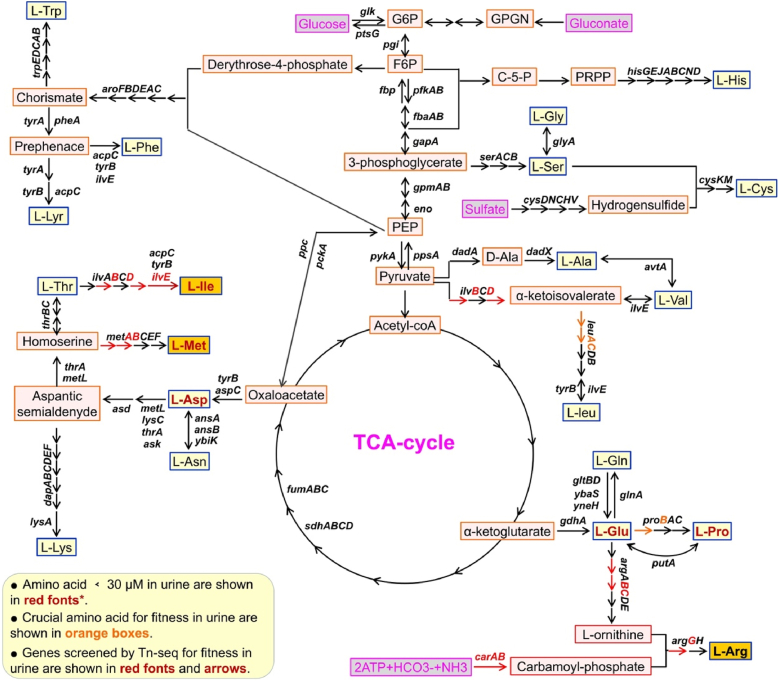
**Overview of candidate fitness genes in the amino acid biosynthesis network of *K. pneumoniae* strain Bckp021 during growth in urine.** Fitness genes were identified by Tn-seq analysis and are highlighted in red font and arrows in the diagram. The diagram shows the roles of 13 candidate fitness genes from *K. pneumoniae* strain Bckp021. Normal concentrations of amino acids in human urine were referenced from the latest research studies [50,51]. (For interpretation of the references to color in this figure legend, the reader is referred to the Web version of this article.)

Nutrient restriction-induced growth inhibition is one of the key factors that induce bacterial biofilm formation. To achieve a sufficient saturation density for identifying *K. pneumoniae* genes essential for optimal growth in urine, a genome-saturating mini-Tn5 transposon mutant library was generated and subsequently used as the inoculum (input) for urine culture under microaerobic condition (2.5 % oxygen) with 50 rpm shaking. After 12 h of incubation at 37 °C, bacterial pellets from urine cultures (output) were harvested. Bacterial genomic DNA was extracted, and transposon insertion sites in both input and output samples were determined using transposon-insertion sequencing (Tn-seq). The Tn-seq reads were then mapped to the Bckp021 genome (comprising a chromosome and five plasmids, CP050834.1 to CP050839.1) to identify the inserted genes. For each gene identified, a fitness score was determined by comparing the ratio of sequencing reads in the output library to those in the input library. A fitness score ≤0.5, with a *P*-value ≤0.05 from three independent assays, indicated that the mutant strain was outcompeted, implying that the disrupted gene plays a role in supporting optimal growth in urine. In total, 19 interrupted genes were identified as essential for bacterial fitness in urine, as their interruption led to a reproducible and substantial decrease in growth (Table S2). Notably, 13 of these genes are involved in amino acid metabolism. Since urine is a relatively nutrient-poor matrix with extremely low protein and amino acid concentrations [21,50], we further investigated the role of amino acid metabolism in *K. pneumoniae* urine fitness. A comprehensive analysis of the *de novo* biosynthesis pathways of 20 amino acids, along with their typical concentrations in human urine, was conducted based on previous reports [50,51]. As shown in Fig. 2, the uptake of arginine, methionine, and isoleucine by *K. pneumoniae* is highly restricted in urine. Therefore, *de novo* biosynthesis of these amino acids is required to mitigate starvation and support optimal growth of strain Bckp021.

### 3.3 Amino acid starvation facilitates the biofilm formation of *K. pneumoniae*

**Fig. 3:**
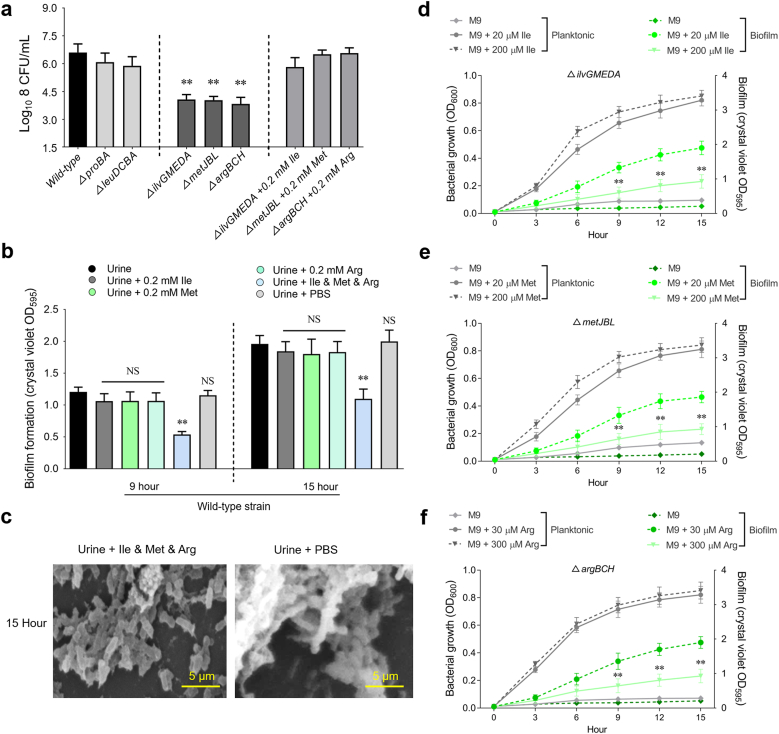
**The roles of arginine, methionine, and isoleucine in the optimal growth and biofilm formation of *K. pneumoniae* in human urine. (a)** Validation of potential urine fitness genes by constructing deletion mutants involved in amino acid biosynthesis. Growth of wild type strain or deletion mutants was quantified by measuring OD_600_ after 8 h incubation at 37 °C in the urine or in the urine with indicated amino acid. (**b)** Biofilm biomass was quantified by measuring absorbance at 595 nm following crystal violet staining of *K. pneumoniae* wild type strain cultured in urine with or without the indicated amino acids at 9 h and 15 h. (**c)** SEM images of *K. pneumoniae* biofilms formed in urine supplemented with Ile&Met&Arg or with PBS, observed at 5000 × magnification. Assays were performed on glass coverslips in the 6-well microtiter plates. (**d-f)** Growth (OD_600_) and biofilm formation (OD_595_) curves of the indicated *K. pneumoniae* mutant strains cultured in M9 medium with or without the indicated amino acids. Values are presented as mean ± SD from three independent experiments. Asterisks denote statistically significant differences in OD_600_ or OD_595_ values between mutant strains and wild-type strains (∗∗*P* < 0.05). (For interpretation of the references to color in this figure legend, the reader is referred to the Web version of this article.)

To assess the involvement of specific genes in the fitness of *K. pneumoniae* in urine, we generated non-polar deletion mutants. Supplementation with 0.2 mM of the corresponding amino acids in urine fully restored the growth of Δ*ilvGMEDA*, Δ*metJBL*, and Δ*argBCH* mutants to levels comparable to the wild-type strain (Fig. 3a). Based on the above data, we hypothesize that bacterial cells may reduce their growth rate in response to arginine, methionine, and isoleucine starvation, which in turn facilitates biofilm formation. As shown in Fig. 3b, supplementation with all three amino acids together, but not with any one of arginine, methionine, or isoleucine at 0.2 mM, significantly decreased biofilm formation of wild-type strain, as indicated by reduced OD_595_ values in urine. Meanwhile, we showed the OD_600_ values at this time point in Fig. S3, which indicated that there are no significant differences among the various strains. This suggested that the amino acid starvation caused by any one of arginine, methionine, and isoleucine was still sufficient to promote the biofilm formation in *K. pneumoniae* strain Bckp021. Consistently, we obtained the same conclusion in two additional *K. pneumoniae* strains, Bckp067 and Bckp091 (Fig. S4). These results strengthened the generality of our findings across multiple isolates. SEM analyses further verified the above result of OD_595_ values (Fig. 3c). To further explore this observation, we assessed the growth and biofilm formation kinetics of the Δ*ilvGMEDA* mutant in M9 medium. Disruption of the *ilvGMEDA* gene, responsible for the *de novo* biosynthesis of isoleucine, resulted in the complete inhibition of bacterial growth when glucose was the sole carbon source. Notably, supplementing the medium with 20 or 200 μM isoleucine effectively restored the growth of the Δ*ilvGMEDA* mutant to levels comparable to the wild-type strain. (Fig. 3d). We observed that biofilm formation in cultures supplemented with 20 μM isoleucine (the normal concentration found in human urine [50,51]) was significantly higher than that in cultures supplemented with 200 μM isoleucine. Similar results were also observed in the growth and biofilm formation kinetics assays of Δ*metJBL* and Δ*argBCH* mutants (Fig. 3e and f). The study suggests that the normal concentrations of arginine, methionine, and isoleucine in human urine are insufficient for *K. pneumoniae* strain Bckp021, leading to starvation of these amino acids and subsequently facilitating biofilm formation.

### 3.4 Amino acid starvation modulates c-di-GMP signaling to facilitate biofilm formation

**Fig. 4:**
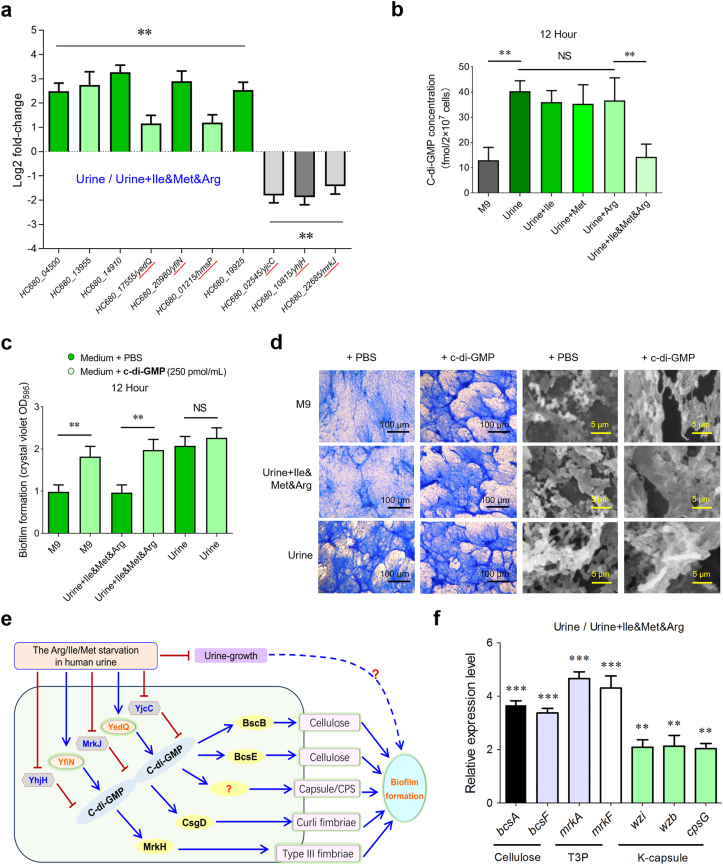
**The roles of amino acid starvation regulating c-di-GMP signaling and biofilm formation during the growth of *K. pneumoniae* in urine. (a)** RT-qPCR was performed to assess the expression levels of 10 c-di-GMP-associated genes, comprising 7 upregulated and 3 downregulated genes, as identified through RNA-Seq analysis. Each experiment was conducted in triplicate, with data normalized against the housekeeping gene *gapA*. Relative expression changes were determined using the 2^−ΔΔCT^ method. **(b)** Measurement of intracellular c-di-GMP levels by LC-MS/MS in *K. pneumoniae* cultured in the indicated media. Error bars indicate the mean ± SD from three biologically independent samples. **(c)** Biofilm formation quantified by OD_595_ measurements after crystal violet staining of *K. pneumoniae* cultured in the indicated media. Data represent means ± SD of triplicate samples. **(d)** Images of biofilms formed by *K. pneumoniae* under different culture conditions. The results were analyzed as described in Fig. 1e. **(e)** Proposed model of c-di-GMP regulation of *K. pneumoniae* biofilm formation in response to amino acid starvation during growth in urine. Biofilm control is depicted as a multilateral coordination module within the c-di-GMP response network referred to the latest research reports [33,35,36,53,[55], [56], [57], [58]]. Blue arrows denote activation, while red T-bar lines denote inhibition. **(f)** The upregulation of cellulose, type III pili (T3P), and K-capsule in *K. pneumoniae* under amino acid starvation in urine. RT-qPCR data were analyzed as described in Fig. 4a. Statistical significance was assessed using two-way ANOVA, comparing mutant strains to the wild-type strain cultured in LB medium (∗∗*P* < 0.01, ∗∗∗*P* < 0.001). (For interpretation of the references to color in this figure legend, the reader is referred to the Web version of this article.)

To identify the molecular mechanisms underlying biofilm formation in strain Bckp021 during amino acid starvation in urine, we conducted a transcriptomic analysis comparing bacterial cells cultured in urine and urine supplemented with arginine, methionine, and isoleucine. Using a stringent threshold (*P* ≤ 0.01) and a minimum two-fold change criterion, we identified numerous genes that were significantly upregulated or downregulated. c-di-GMP functions as a key intracellular signal that enables bacteria to cope with amino acid deprivation [25,26] and is closely linked to diverse biological processes and biofilm formation in *K. pneumoniae* [52]. Here a total of 30 c-di-GMP signaling related genes were detected in the above transcriptomic data (Table 1), including 13 diguanylate cyclase (DGC) genes involved in c-di-GMP synthesis, 11 phosphodiesterases (PDE) genes responsible for c-di-GMP degradation, and six genes encoding both enzymatic domains. Among them, eight DGC genes and four DGC + PDE genes were significantly upregulated, while four PDE genes were significantly downregulated. To verify the transcriptomic results, RT-qPCR was performed to check the expression of 10 selected genes. The relative expression levels of seven upregulated genes (including *yedQ* and *yfiN*) and three downregulated genes (including *yjcC*, *mrkJ* and *yhjH*) were consistent with transcriptomic data, confirming the reliability of our results (Fig. 4a).

**Table 1:** Differential expression genes (Urine/Urine + Ile&Met&Arg at least twofold, *P* < 0.01) involved in c-di-GMP signaling.

Locus_tag/gene	Ref.	GGDEF (DGC)	EAL (PDE)	Other domain	Log2 fold-change
*HC680_02065*		**✓**		*Cache_1*	1.258
*HC680_04500*		**✓**		–	**2.121**
*HC680_09460*		**✓**		–	0.312
*HC680_10720*^*a*^		**✓**		*MASE2*	−0.089
*HC680_10835*		**✓**		*HAMP, GAF*	0.931
*HC680_10885*		**✓**		*GAF*	1.236
*HC680_13055*		**✓**		–	1.023
*HC680_13955*		**✓**		*PAS_4*	**2.563**
*HC680_14910*		**✓**		–	**3.126**
*HC680_17555/yedQ/dgcQ*	[53]	**✓**		*Cache_1*	**1.062**
*HC680_18085*		**✓**		*GAF*	−0.041
*HC680_20980/yfiN*	[53]	**✓**		*HAMP*	**2.712**
*HC680_28165*		**✓**		–	0.539
*HC680_01215/hmsP*	[54]	**✓**	**✓**	*GAPES3*	**1.235**
*HC680_13280*		**✓**	**✓**	*PAS*	0.946
*HC680_18120*		**✓**	**✓**	–	0.121
*HC680_19925*		**✓**	**✓**	*MASE2*	**2.515**
*HC680_20360*		**✓**	**✓**	*MASE1*	1.425
*HC680_24700*		**✓**	**✓**	*GAPES4*	1.369
*HC680_02545/yjcC*	[55]		**✓**	*CSS-motif*	**−1.504**
*HC680_06905*			**✓**	*CSS-motif*	−0.775
*HC680_08945*			**✓**	*BLUF*	0.727
*HC680_10725*^*a*^			**✓**	–	0.572
*HC680_10815/yhjH*	[56]		**✓**	–	**−1.513**
*HC680_12795*			**✓**	*BLUF*	0.619
*HC680_16820*			**✓**	*CSS-motif*	0.094
*HC680_22685/mrkJ*	[33]		**✓**	–	**−1.31**
*HC680_22785*			**✓**	–	0.683
*HC680_26745*			**✓**	–	−0.735
*HC680_28790*			**✓**	–	−0.715

Given the observed upregulation of DGC genes and downregulation of PDE genes, it was reasonable to expect a significant increase in c-di-GMP synthesis. To confirm this, we measured intracellular c-di-GMP levels under different culture conditions. The results showed significantly higher c-di-GMP levels in amino acid-starved cultures (urine, urine + Ile, urine + Met and urine + Arg) compared to M9 and urine + Ile&Met&Arg cultures (Fig. 4b), suggesting a relationship between the upregulated c-di-GMP signaling and increased biofilm formation under amino acid starvation conditions. As shown in Fig. 4c, supplementation with exogenous c-di-GMP in M9 medium or urine + Ile&Met&Arg significantly promoted *K. pneumoniae* biofilm formation to levels comparable to those observed in urine cultures. Microscopy analyses using crystal violet staining and SEM further supported these observations (Fig. 4d), aligning with OD_595_ measurements and reinforcing the role of c-di-GMP in facilitating *K. pneumoniae* biofilm formation in urine. Previous studies have confirmed that YedQ, YfiN, YjcC, MrkJ and YhjH regulate intracellular c-di-GMP levels [53,55,56]. Additionally, c-di-GMP signaling positively regulates BcsB and BcsE for cellulose synthesis [36], CsgD for curli fimbriae assembly [56,57], and MrkH for type III fimbriae assembly [35]. Cellulose, curli fimbriae, type III fimbriae, and the capsule are all well-characterized in their roles promoting bacterial biofilm formation [58,59]. Based on these findings, we proposed a regulatory network in which c-di-GMP signaling mediates biofilm formation in response to amino acid starvation in urine (Fig. 4e). To further validate this model, we assessed the expression levels of genes involved in extracellular matrix production. The expression of *bcsA* and *bcsF* (cellulose synthesis), *mrkA* and *mrkF* (type III fimbriae synthesis), and *wzi*, *wzb* and *cpsG* (capsule synthesis) was significantly upregulated in urine compared to urine + Ile&Met&Arg cultures (Fig. 4f), providing additional support for our proposed regulatory network.

### 3.5 The EPS biosynthesis network of *K. pneumoniae* is activated for biofilm formation in urine

**Fig. 5:**
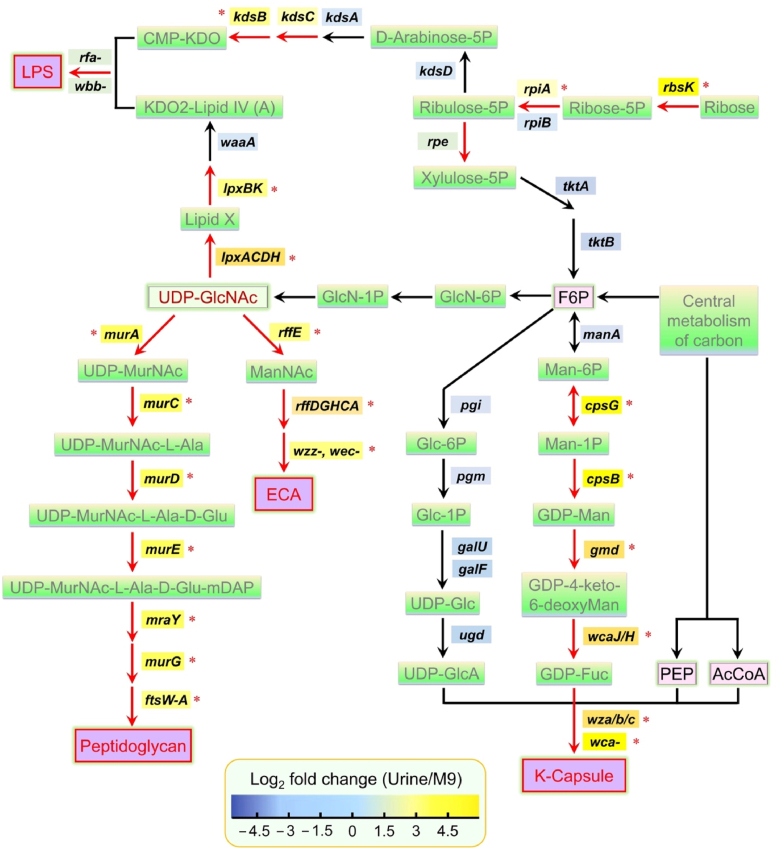
**EPS biosynthesis gene network in *K. pneumoniae* is upregulated in response to urine under low oxygen conditions.** Gene expression differences between *K. pneumoniae* cultured in urine versus M9 medium were analyzed. Samples from three independent biological replicates were collected after 12 h of incubation for transcriptomic analysis. Genes exhibiting more than a twofold increase in expression with *P* < 0.05 were considered significantly upregulated. Log_2_ fold-change values were visualized using a divergent color gradient. F6P, fructose-6-phosphate; PEP, phosphoenolpyruvate. (For interpretation of the references to color in this figure legend, the reader is referred to the Web version of this article.)

**Fig. 6:**
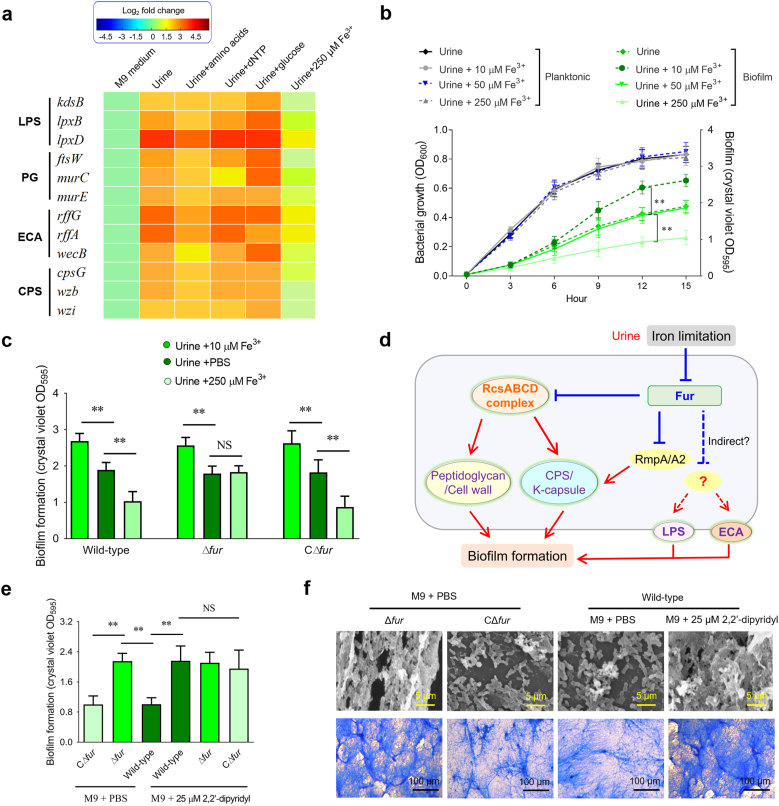
**The roles of iron limitation regulating EPS biosynthesis and biofilm formation during the growth of *K. pneumoniae* in urine. (a)** Iron limitation in urine induces the upregulation of genes involved in EPS biosynthesis. A heat map was generated to visualize the relative transcriptional alterations of selected genes. RT-qPCR was employed to assess gene expression following 12 h of incubation under specified conditions. Data from three independent biological replicates were subjected to two-way ANOVA analysis. **(b)** Growth (OD_600_) and biofilm formation (OD_595_) curves of *K. pneumoniae* cultured in urine with or without Fe^3+^ supplementation at varying concentrations. **(c)** Quantification of biofilm formation (OD_595_) stained with crystal violet in wild-type and mutant strains cultured in the indicated media. Data represent the mean ± SD from triplicate samples. **(d)** Proposed regulatory model of the global regulator Fur in *K. pneumoniae* biofilm formation under iron-limited conditions during growth in urine. Biofilm regulation is illustrated as a complex, coordinated network within the Fur regulatory system, based on recent studies [[60], [61], [62], [63], [64]]. Positive regulation is depicted by red arrows, while blue lines with flat ends represent negative regulation. **(e)** Biofilm quantification (OD_595_) in wild-type and mutant strains cultured in M9 medium with or without 2,2′-dipyridyl. Iron-depleted medium was prepared by supplementing M9 with 25 μM 2,2′-dipyridyl. Values are presented as mean ± SD from triplicate samples. **(f)** Images of biofilms formed by wild-type and mutant strains cultured under the indicated conditions. The results were analyzed as described in Fig. 1e. (For interpretation of the references to color in this figure legend, the reader is referred to the Web version of this article.)

To further investigate the potential mechanisms underlying *K. pneumoniae* strain Bckp021 biofilm formation, we compared its transcriptome when cultured in urine versus M9 medium. Using a 5 % false-discovery rate threshold and *q-*value cutoff of 0.10, we identified more than 100 genes that were significantly upregulated, at least twofold under urine-simulated growth conditions. As expected, genes related to iron uptake and metabolism were among those upregulated. Notably, we also observed significant upregulation of numerous genes involved in exopolysaccharides (EPS) biosynthesis, which have been previously reported to contribute to bacterial biofilm formation [3,16], were also upregulated in this study (Table S3). Most of the identified genes occupy key positions within the bacterial polysaccharide metabolic network (Fig. 5), and can be categorized into several biosynthesis clusters, including those for LPS (*lpxACDH*, *lpxBK*, *kdsB* and *kdsC*), peptidoglycan (*murA*, *murEmraY*, *murD*, *ftsW-murGC*, *ftsA*), enterobacterial common antigen (ECA) (*wzzE*, *rffE-wecC*, *rffDGHCA*, *wecFG*), and K-capsule (*cpsG*, *cpsB*, *gmd*, *wcaJ/H*, wza/b/c, *wca*). To validate the transcriptomic data, RT-qPCR was performed to assess the expression of key genes from these biosynthetic pathways. We selected 12 representative genes (*kdsB*, *lpxB*, *lpxD*, *ftsW*, *murC*, *murE*, *rffG*, *rffA*, *wecB*, *cpsG*, *wzb* and *wzi*) spanning the four major EPS biosynthesis pathways: LPS, peptidoglycan, ECA, and K-capsule. RNA was extracted from bacterial cells cultured in urine for 12 h (approximately 10^8^ CFU/mL) and analyzed via RT-qPCR. The results demonstrated significant upregulation of all selected genes in urine compared to M9 cultures (Fig. 6a), reinforcing their potential roles in biofilm formation under these conditions.

### 3.6 *K. pneumoniae* increase EPS biosynthesis for biofilm formation in response to the iron limitation of urine

We next investigated whether amino acid starvation regulates the EPS biosynthesis network through c-di-GMP signaling. However, supplementation with a mixture of 20 amino acids in urine did not change gene expression levels (Fig. 6a). Similar results were observed when urine was supplemented with glucose and dNTP. In contrast, supplementation with excess Fe^3+^ ions significantly inhibited the urine-induced upregulation of these genes, reducing their expression to levels comparable to those observed in M9 cultures (Fig. 6a). Given the role of EPS biosynthesis in biofilm formation and the iron-limited nature of urine, we examined the growth and biofilm formation kinetics of *K. pneumoniae* in urine supplemented with 10, 50, and 250 μM Fe^3+^ ions. As shown in Fig. 6b, supplementation with 10 μM Fe^3+^ ions significantly enhanced biofilm formation, consistent with previous studies demonstrating that iron positively regulates biofilm formation in *K. pneumoniae* [15]. We observed that supplementation with 50 μM Fe^3+^ ions resulted in biofilm formation levels similar to those observed in urine alone, while 250 μM Fe^3+^ ions significantly suppressed biofilm formation (Fig. 6b). Importantly, the same pattern was observed in two additional clinical isolates, Bckp067 and Bckp091 (Fig. S4), which broadened the applicability of our findings. These findings suggested that an optimal concentration of iron ions promotes biofilm formation, whereas excessive iron exerts an inhibitory effect.

Fur, an iron-responsive global regulator, has been shown to influence biofilm formation in *K. pneumoniae* [18,20]. In this study, the *fur* deletion mutant showed a similar level of biofilm formation with the wild-type strain, indicating that the Fur-mediated repression is relieved in urine (Fig. 6c). All three strains (wild-type, Δ*fur*, and CΔ*fur*) showed increased biofilm formation in urine supplemented with 10 μM Fe^3+^ ions, suggesting that this phenotype was independent of Fur regulation and that the supplementation of 10 μM Fe^3+^ ions did not alleviate Fur-mediated repression of downstream genes. In contrast, Fur-mediated repression was activated when urine was supplemented with 250 μM Fe^3+^ ions, leading to a significant downregulation in biofilm formation. However, in the *fur* deletion mutant, this repression was relieved, restoring biofilms formation to levels comparable to those observed in urine alone (Fig. 6c). Previous studies have confirmed the Fur regulates both the Rcs signal transduction system and the RmpA/A2 regulator [60,61], both of which positively regulate K-capsule synthesis, thereby facilitating biofilm formation [62,63]. Additionally, Fur-mediated regulation of LPS, ECA, and peptidoglycan synthesis has been well studied in its roles in promoting bacterial biofilm formation [16,64]. Based on these findings, we proposed a potential regulatory network in which Fur modulates biofilm formation in iron-limited urine (Fig. 6d). To further verify the above findings, we examined biofilm formation in M9 medium with or without 2,2′-dipyridyl (an iron chelator). As shown in Fig. 6e, deletion of *fur* significantly enhanced biofilm formation in M9 medium compared to the wild-type and complementary strains. Moreover, supplementation with 2,2′-dipyridyl alleviated Fur-mediated repression, increasing biofilm formation in the wild-type and complementary strains to levels comparable to those observed in the Δ*fur* mutant. Crystal violet-stained slide microscopy and SEM analyses further confirmed these observations (Fig. 6f), consistent with the results of OD_595_ values. Furthermore, we also compared the transcriptional levels of genes related to cellulose, T3P, *csgD* and K capsule after the addition of Fe^3+^. It was found that only the transcriptional level of the K capsule gene significantly increased (Fig. S5). All these findings suggested that Fur controlled the biofilm formation of *K. pneumoniae* in urine by regulating the EPS biosynthesis network.

## 4 Discussion

Biofilm formation is widely recognized as a key factor enabling the persistence of *K. pneumoniae*, but its exact role in disease causation remains incompletely defined, in part because available animal models do not yet fully reproduce human urinary tract conditions. *Ex vivo* cultivation in human urine offers a convenient proxy that captures certain aspects of the urinary milieu, but coupling urine-based assays with complementary approaches would better emulate the temporal and spatial complexity of natural infections. Genome-scale transposon screens and transcriptional profiling performed for UPEC in urine have uncovered multiple fitness and virulence pathways linked to urinary tract colonization, suggesting that analogous strategies could reveal urine-adaptation determinants in *K. pneumoniae*. Identifying such urine-responsive genes would help clarify mechanisms behind catheter-associated biofilm development and other device-related infections. In the present work, we found that amino acid starvation (AAS) and iron limitation act as major modulators of biofilm formation by *K. pneumoniae* in the nutrient-limited environment of urine; by contrast, although biofilms also form in nutrient-replete conditions, the relative abundance of biofilm versus planktonic cells is reduced under those conditions. These findings highlight that biofilm formation is not merely a passive response to stress, but a strategic adaptation deployed when key nutrients become limiting. Given this nutrient-dependent phenotype, it is important to consider the biochemical characteristics of urine. Urine is intrinsically nutrient-poor, containing low levels of amino acids and carbohydrates but relatively high concentrations of metabolic waste products. Although the absolute concentrations of individual amino acids vary, previous quantitative analyses indicate that these differences are not substantial enough to explain why *K. pneumoniae* experiences marked starvation specifically for arginine, methionine, and isoleucine [51,65]. Therefore, the enhanced biofilm formation observed under limitation of these three amino acids is unlikely to result simply from their unusually low abundance in urine. Instead, our data suggest that *K. pneumoniae* may have disproportionately higher metabolic requirements for arginine, methionine, and isoleucine during growth in urine. This interpretation is supported by observations in uropathogenic *E. coli*, which exhibits similar auxotrophic constraints for these amino acids when proliferating in urine [21]. Together, these considerations indicate that pathogen-specific metabolic demands—rather than absolute nutrient concentrations alone—shape the starvation responses that drive biofilm formation in the urinary tract.

c-di-GMP functions as a central bacterial second messenger that mediates responses to AAS and orchestrates the switch between free-living and surface-attached states, thereby governing biofilm development [66]. In *K. pneumoniae*, production of EPS and expression of type-3 fimbrial/pilus (T3P) components are major determinants of biofilm formation [16], and both processes are under the influence of intracellular c-di-GMP concentrations. Elevated cellular c-di-GMP favors matrix production and fimbrial expression, promoting sessility, whereas reduced c-di-GMP is associated with increased motility and biofilm dispersal [35,67]. High c-di-GMP levels have been shown to suppress motility while stimulating EPS synthesis and biofilm formation [68]; conversely, low levels correlate with enhanced locomotion and diminished adhesion and matrix output [69]. Genome-wide surveys of GGDEF/EAL domain–containing proteins in *K. pneumoniae* reveal that most loci are conserved across strains though certain genes are strain-specific [32]. The presence of both single-domain GGDEF proteins and hybrid GGDEF–EAL architectures, together with considerable copy number variation, points to a multilayered and sophisticated c-di-GMP regulatory network in this species, highlighting the importance of multiple regulatory mechanisms for optimal bacterial survival. Nevertheless, only a subset of these signaling components has been characterized functionally, leaving the roles of many GGDEF/EAL proteins to be elucidated.

Characterization of the *K. pneumoniae* biofilm matrix indicates that extracellular polysaccharides are the dominant constituents, with both lipopolysaccharide and capsular polysaccharide components contributing substantially to the structural matrix [5,6]. The capsule influences multiple stages of biofilm development — from the earliest surface attachment to later maturation processes — although its net effect varies with strain background and capsule type [70]. For example, removal of the capsule enhanced biofilm formation in a hypermucoviscous K1 isolate but produced the opposite effect in a K124 isolate, implying that both adhesive structures and the amount or composition of capsule jointly determine biofilm outcomes [9]. LPS is a crucial component of the outer membrane in Gram-negative bacteria. It plays a significant role in the initial attachment to non-living surfaces and is essential during the early stages of biofilm development [70]. While some LPS biosynthetic and transport loci (e.g., *wbbM* and *wzm*) have been reported to show elevated expression in *K. pneumoniae* biofilm-grown cells relative to exponential-phase planktonic cultures [71]. In this study, diverse genes associated with LPS and the K-capsule were identified to be significantly upregulated in bacterial cells growth in urine (strong biofilm formation) comparison with in M9 medium (Less biofilm formation). However, the expression of *wbbM* and *wzm* did not show significant changes. Instead, we observed broad upregulation of genes linked to peptidoglycan assembly and the enterobacterial common antigen pathway — two additional EPS-related systems — among bacteria grown in urine, consistent with activation of a wider EPS biosynthetic program under nutrient-limited conditions.

Iron limitation functions as a key environmental cue that promotes bacterial colonization and infection following contact with host tissues. In *K. pneumoniae*, iron homeostasis is principally controlled by the ferric uptake regulator Fur [18], which, when bound to iron, represses iron-acquisition systems under iron-replete conditions and is derepressed in iron-poor host niches. Paradoxically, transcriptomic studies have shown that siderophore biosynthetic loci are markedly downregulated in biofilm-dispersed and sessile *K. pneumoniae* populations relative to planktonic cells [72], a change that has been associated with decreased siderophore output and speculated to attenuate host immune activation [73]. At the same time, some liver-abscess–associated isolates display enhanced biofilm formation when cultured with supplemental iron, whereas growth and biofilm development are impaired by the iron chelator 2,2′-dipyridyl, indicating strain- and context-dependent effects of iron on *K. pneumoniae* physiology [15]. These apparently discordant observations underscore the complexity of the iron–biofilm regulatory axis *in vivo* and mirror the variable influence of the polysaccharide capsule on biofilm phenotypes, which can either favor or hinder biofilm assembly depending on an isolate's hypermucoviscous characteristics. Moreover, Fur has been implicated in promoting fimbrial gene expression under iron-replete conditions — including type I [18,19] and type III fimbrial loci [20] and their transcriptional regulators — thereby linking iron sensing to adhesive apparatus regulation [18,20]. In our experiments, however, T3P biosynthesis in *K. pneumoniae* during urinary tract–related growth appears to be more tightly controlled by c-di-GMP signaling, while EPS biosynthetic responses to urine's iron scarcity are predominantly Fur-dependent. Comparable regulatory roles for Fur in modulating EPS pathways have been reported in extraintestinal pathogenic *E. coli* (ExPEC) during bloodstream infection under iron-limited conditions [74]. Finally, the observation that *K. pneumoniae* biofilm formation can be substantially reduced when EPS is targeted (for example, by depolymerase-encoding bacteriophages) and in the presence of divalent Co^2+^ ions (an iron-antagonizing ion) supports the functional linkage between Fur-mediated iron sensing, EPS metabolism, and biofilm integrity [75]. Although low amino acid availability and iron limitation primarily influence biofilm formation through distinct pathways, their shared regulation of specific extracellular components—most notably the K capsule—suggests that these cues may not act independently but instead exhibit a degree of crosstalk. Importantly, analysis of the regulatory networks indicated that CsgD is not part of the iron-limitation response, implying that the two signals do not jointly downregulate this master regulator. Consistent with this, Fe^3+^ supplementation restored the transcription of K-capsule genes alone, further supporting that only select extracellular polymers are co-regulated by both cues. Given that *K. pneumoniae* strains are highly diverse, these factors provide a basis for further investigation across a broader range of isolates to determine how universal these physiological cues are among different strains.

Further research on establishing an *ex vivo* model of urinary catheters to investigate the influence of flowing urine on the biofilm formation will enable a more accurate reflection of the characteristics of *K. pneumoniae* biofilm during catheter-associated infections and UTIs. In natural settings, biofilms typically form as multispecies consortia rather than single-species layers, and mixed-species interactions have been implicated in device-associated urinary infections that originate from biofilm communities on urologic implants [76]. Surveys of culture-positive urinary catheters commonly report two or more bacterial taxa per sample, with *Pseudomonas aeruginosa*, *E. coli* and *K. pneumoniae* frequently recovered together [76]. Experimental work indicates that *K. pneumoniae*'s AI-2 uptake systems can shape interspecies dynamics and help determine species balance and biomass in tri-member biofilm assemblages [77]. Increasing evidence therefore indicates that microbes colonizing medical devices or host surfaces coexist and interact cooperatively within polymicrobial biofilms [78,79], and that such community-level interactions often increase collective tolerance to host immune effectors and to antimicrobial therapies across the constituent species [80,81]. Elucidating the molecular and ecological bases of these polymicrobial interactions remains essential to understand persistence on devices and to develop strategies that disrupt community-mediated resistance.

## 5 Conclusions

In summary, our findings revealed a sophisticated interplay between environmental nutrient limitations and regulatory pathways that collectively promote *K. pneumoniae* biofilm formation in the urinary tract. Specifically, we identified amino acid biosynthesis and iron acquisition as key metabolic processes essential for bacterial fitness under urine-specific conditions. These nutrient constraints activated downstream responses, including the modulation of c-di-GMP signaling and the induction of exopolysaccharide biosynthesis, thereby enhancing biofilm development. By integrating genetic, transcriptomic, and phenotypic data, this study offers a systems-level perspective on how *K. pneumoniae* adapts to the urinary environment. Collectively, these insights expand our knowledge of UTI pathogenesis and highlight potential molecular targets for the development of anti-biofilm strategies against persistent or catheter-associated infections. However, *K. pneumoniae* exhibits considerable strain-to-strain variability, additional studies involving diverse isolates will be essential to determine the generalizability of these regulatory mechanisms.

## CRediT authorship contribution statement

**Xinming Pan:** Writing – original draft, Visualization, Validation, Methodology, Investigation, Formal analysis, Data curation, Conceptualization. **Yinchu Zhu:** Visualization, Validation, Methodology, Investigation, Formal analysis, Data curation, Conceptualization. **Yan Zhang:** Validation, Methodology, Investigation, Formal analysis. **Jie Zhao:** Validation, Methodology, Investigation, Formal analysis. **Xing Gao:** Visualization, Validation, Investigation. **Caiying Li:** Visualization, Validation, Methodology, Investigation. **Yong Yu:** Writing – review & editing, Resources, Methodology. **Jiale Ma:** Writing – review & editing, Supervision, Project administration, Funding acquisition, Data curation, Conceptualization.

## Ethics approval and consent to participate

The healthy human urine samples were collected from 30 volunteers including men and women of different ages, which were used solely for *in vitro* experiments. The study was approved by the Institutional Review Board (IRB ID: 24–171) of the Affiliated Hospital of Yangzhou University, in accordance with the policies of the Medicine Human Subjects Committee of Jiangsu Province. Written permission for urine collection was secured from human subjects and/or their legal representatives.

## Availability of data and materials

All raw data associated with this work are available via Figshare (https://doi.org/10.6084/m9.figshare.28787327). The chromosome and plasmids information of *K. pneumoniae* strain Bckp021 have been uploaded in the NCBI GenBank database under accession numbers CP050834.1 to CP050839.1.

## Funding

This research was supported by the 10.13039/501100012166National Key Research and Development Program of China (2023YFD1800503), and the 10.13039/501100001809National Natural Science Foundation of China (31802187), and the Open Project Program of Jiangsu Key Laboratory of Zoonosis (No. 2310).

## Declaration of competing interest

The authors declare that they have no known competing financial interests or personal relationships that could have appeared to influence the work reported in this paper.

## Data Availability

Data will be made available on request.
