# Supplementary material for: Amino acid starvation and iron limitation facilitate the biofilm formation of Klebsiella pneumoniae within urine
Source: Biofilm. 2026 Jan 16;11:100347. doi: 10.1016/j.bioflm.2026.100347 (PMC12857406; doi:10.1016/j.bioflm.2026.100347)
Supplement: Multimedia component 1 [file mmc1.docx]

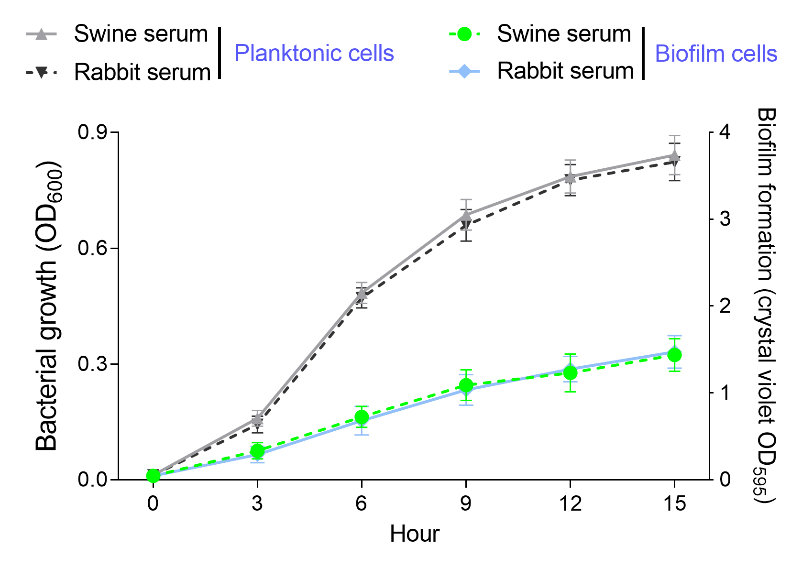


**Figure S1** The growth (OD_600_) and biofilm formation (OD_595_) curves of *K. pneumoniae* strain Bckp021 cultured in swine serum and rabbit serum. Values are presented as mean ± SD from three independent experiments.


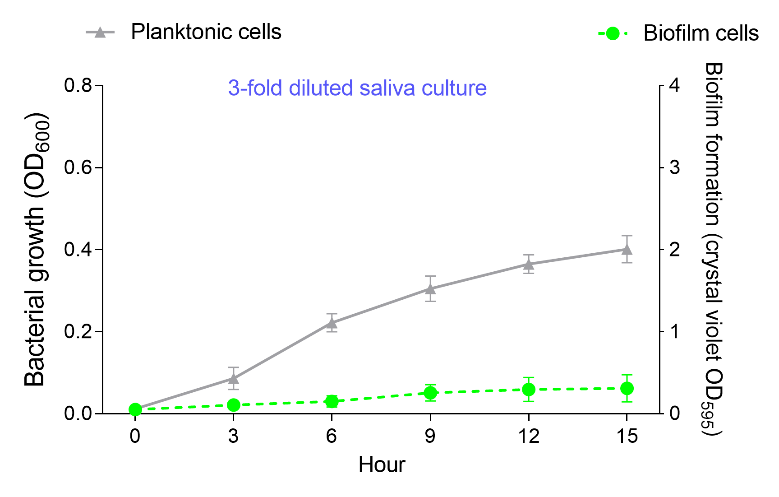


**Figure S2** The growth (OD_600_) and biofilm formation (OD_595_) curves of *K. pneumoniae* strain Bckp021 cultured in 3-fold diluted saliva culture. Values are presented as mean ± SD from three independent experiments.


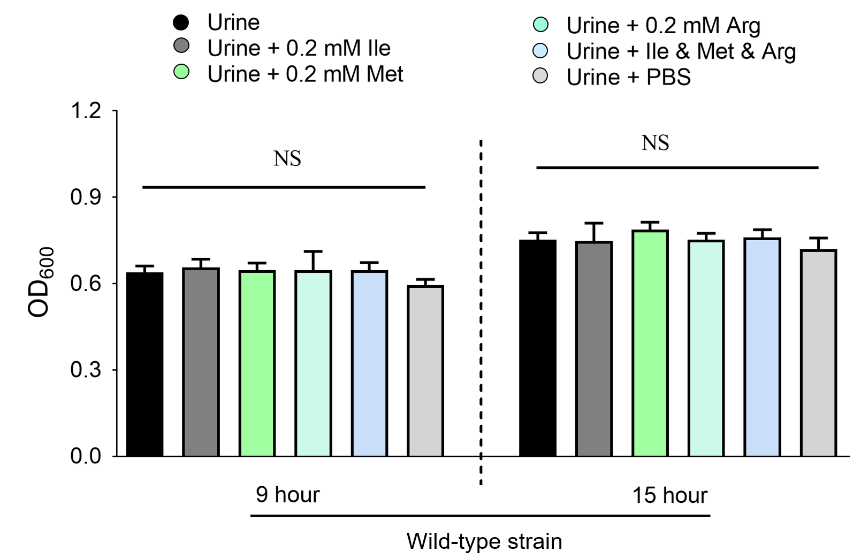


**Figure S3** The growth of *K. pneumoniae* wild-type strain Bckp021 was quantified by measuring absorbance at 600 nm cultured in urine with or without the indicated amino acids at 9 hour and 15 hour.


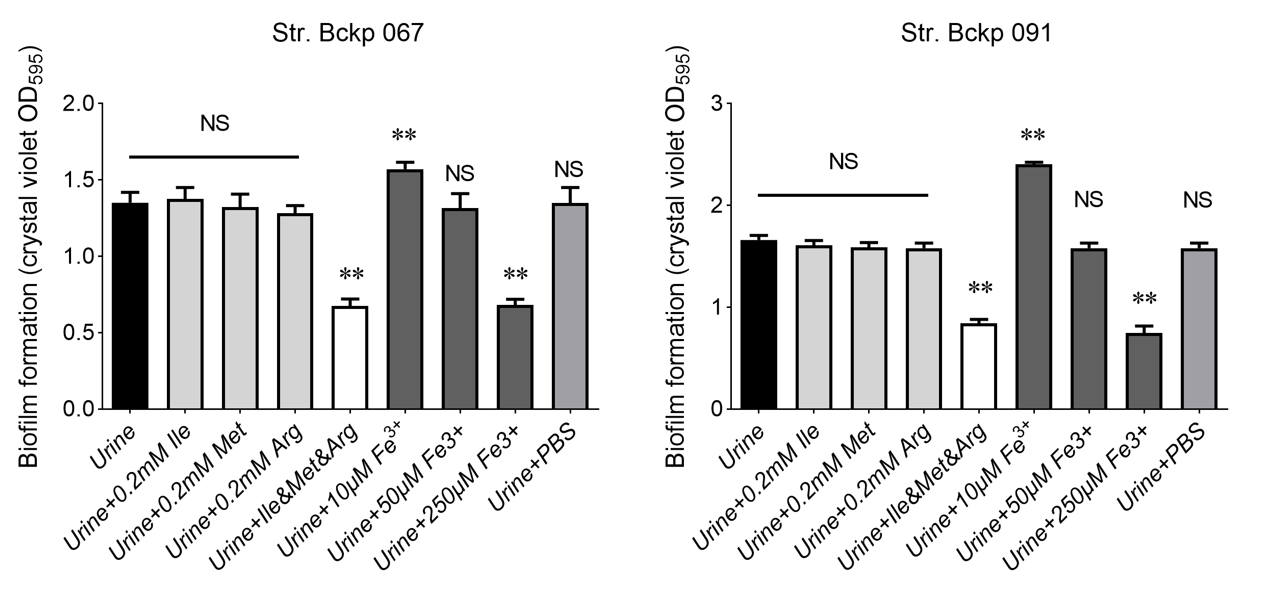


**Figure S4** Biofilm biomass was quantified by measuring absorbance at 595 nm following crystal violet staining of *K. pneumoniae* strains Bckp067 (left) and Bckp091 (right) cultured in urine with or without the indicated amino acids and Fe^3+^ supplementation.


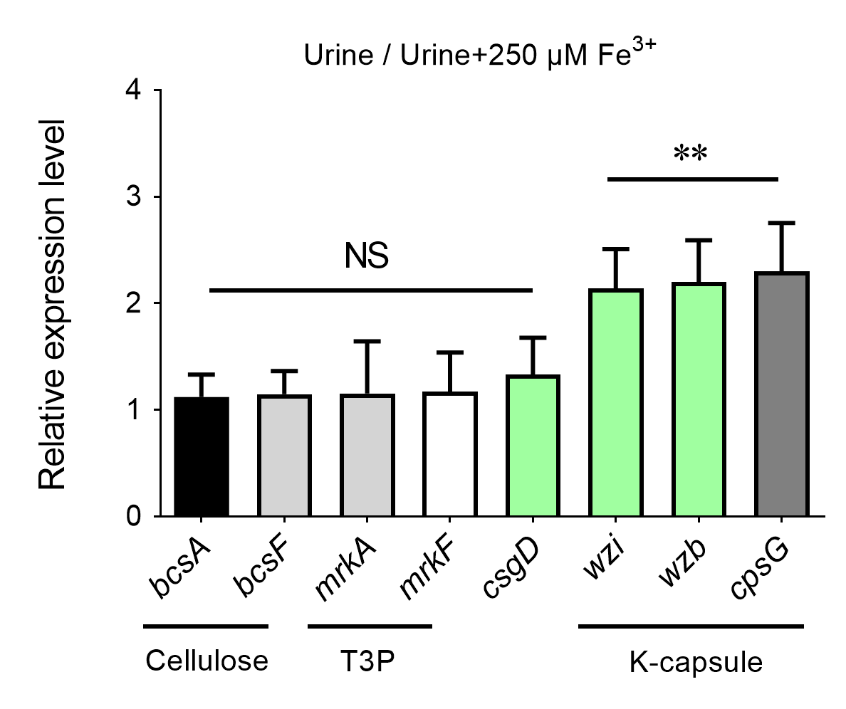


**Figure S5** Expression differences of cellulose, type III fimbriae (T3P), *csgD*, and the K capsule in *K. pneumoniae* strain Bckp021 when grown in urine or in urine supplemented with Fe³⁺.

**Table S1** Bacterial strains and plasmids used in this study.

| **Strains or plasmids** | **Information** | **Source or ref.** |
| --- | --- | --- |
| **Bacterial strains** |  |  |
| Bckp021 | A hypervirulent K2 isolate from the milk  samples possibly associated with an infection incident in children | Our laboratory [1] |
| *ΔilvGMEDA* | Deletion mutant of *ilvGMEDA* with Bckp021 background | This study |
| *ΔmetJBL* | Deletion mutant of *ilvJBL* with Bckp021 background | This study |
| *ΔargBCH* | Deletion mutant of *argBCH* with Bckp021 background | This study |
| Δ*fur* | Deletion mutant of *fur* with Bckp021 background | This study |
| CΔ*fur* | Replenished of *fur* using pGEN-pcm with Δ*fur* background | This study |
| *E. coli* DH5α | Cloning host for maintaining the recombinant plasmids | Invitrogen |
| **Plasmid** |  |  |
| pGEN-pcm | *E. coli* shuttle vector pGEN MCS with a Pcm promoter; Amp^R^ | Laboratory stock |
| pGEN-*fur* | pGEN MCS carrying *fur* under the control of Pcm promoter | This study |
| pKD46 | Red recombinase expression plasmid | [2] |
| PKD4 | pANTS derivative containing FRT-flanked kanamycin resistance | [2] |
| pCP20 | TS replication and thermal induction of FLP synthesis | [2] |

**Table S2** Fitness factors (quantity ratio of output/input reads under the urine culture) ≤ 0.5 (*P* ≤ 0.05 of three repeat assays).

| Gene | Fitness factor | *P*-value |
| --- | --- | --- |
| *carA* | 0.211 | 0.0046 |
| *carB* | 0.265 | 0.0057 |
| *proB* | 0.496 | 0.0178 |
| *argB* | 0.375 | 0.0098 |
| *argC* | 0.366 | 0.0087 |
| *argG* | 0.354 | 0.0093 |
| *metA* | 0.321 | 0.0102 |
| *metB* | 0.296 | 0.0114 |
| *leuA* | 0.465 | 0.0201 |
| *leuC* | 0.423 | 0.0215 |
| *ilvB* | 0.355 | 0.0121 |
| *ilvD* | 0.346 | 0.0144 |
| *ilvE* | 0.276 | 0.0086 |
| *purA* | 0.234 | 0.0054 |
| *guaA* | 0.255 | 0.0032 |
| *guaB* | 0.213 | 0.0029 |
| *HC680_00045* | 0.398 | 0.0118 |
| *HC680_00050* | 0.365 | 0.0156 |
| *HC680_05750* | 0.374 | 0.0178 |

**Table S3** Differential expression genes (urine / M9 at least two-fold, *P*<0.05) involved in EPS biosynthesis.

| Gene | Log_2_ fold-change | *P* value | Function |
| --- | --- | --- | --- |
| *kdsB* | 3.1 | 0.0033 | 3-deoxy-manno-octulosonate cytidylyltransferase |
| *kdsC* | 2.1 | 0.0035 | 3-deoxy-manno-octulosonate-8-phosphatase |
| *rbsK* | 4.2 | 0.0023 | Ribokinase |
| *rpiA* | 2.2 | 0.0035 | Ribose-5-phosphate isomerase |
| *lpxB* | 3.1 | 0.0013 | Lipid-A-disaccharide synthase |
| *lpxK* | 3.2 | 0.0021 | Tetraacyldisaccharide 4'-kinase |
| *lpxA* | 6.2 | 0.0004 | acyl-ACP--UDP-N-acetylglucosamine O-acyltransferase |
| *lpxC* | 5.9 | 0.0003 | UDP-3-O-[3-hydroxymyristoyl] N-acetylglucosamine deacetylase |
| *lpxD* | 6.1 | 0.0006 | UDP-3-O-(3-hydroxymyristoyl) glucosamine N-acyltransferase |
| *lpxH* | 6.3 | 0.0010 | UDP-2,3-diacylglucosamine diphosphatase |
| *murA* | 4.2 | 0.0041 | UDP-N-glucosamine 1-carboxyvinyltransferase |
| *murC* | 4.3 | 0.0032 | UDP-N-acetylmuramate--L-alanine ligase |
| *murD* | 4.1 | 0.0005 | UDP-N-acetylmuramoyl-L-alanine--D-glutamate ligase |
| *murE* | 4.0 | 0.0003 | O-succinylbenzoate-CoA synthase |
| *murG* | 4.3 | 0.0016 | Undecaprenyldiphospho-muramoylpentapeptide beta-N-acetylglucosaminyltransferase |
| *mraY* | 4.4 | 0.0023 | Phospho-N-acetylmuramoyl-pentapeptide-transferase |
| *ftsW* | 4.1 | 0.0024 | Cell division protein FtsW |
| *murD* | 4.2 | 0.0039 | UDP-N-acetylmuramoyl-L-alanine--D-glutamate ligase |
| *murF* | 4.2 | 0.0054 | UDP-N-acetylmuramoyl-tripeptide--D-alanyl-D-alanine ligase |
| *ftsI* | 4.1 | 0.0021 | Peptidoglycan glycosyltransferase FtsI |
| *ftsL* | 4.0 | 0.0035 | Cell division protein FtsL |
| *rffE* | 3.4 | 0.0039 | UDP-N-acetyl glucosamine-2-epimerase |
| *rffD* | 5.8 | 0.0006 | UDP-N-acetyl glucosamine-2-epimerase |
| *rffG* | 5.9 | 0.0003 | dTDP-glucose 4,6-dehydratase |
| *rffH* | 5.9 | 0.0008 | Glucose-1-phosphate thymidylyltransferase |
| *rffC* | 5.5 | 0.0009 | dTDP-4-amino-4,6-dideoxy-D-galactose acyltransferase |
| *rffA* | 5.6 | 0.0016 | dTDP-4-amino-4,6-dideoxygalactose transaminase |
| *wzzE* | 3.1 | 0.0023 | ECA polysaccharide chain length modulation protein |
| *wzxE* | 3.2 | 0.0028 | Lipid III flippase WzxE |
| *wzyE* | 3.0 | 0.0041 | O-antigen assembly polymerase |
| *wecC* | 3.3 | 0.0016 | UDP-N-acetyl-D-mannosamine dehydrogenase |
| *wecB* | 3.3 | 0.0013 | UDP-N-acetylglucosamine 2-epimerase (non-hydrolyzing) |
| *wecA* | 3.2 | 0.0026 | UDP-N-acetylglucosamine--undecaprenyl-phosphate N-acetylglucosaminephosphotransferase |
| *cpsG* | 4.9 | 0.0056 | Phosphomannomutase |
| *cpsB* | 4.9 | 0.0006 | Mannose-1-phosphate guanylyltransferase |
| *gmd* | 6.1 | 0.0002 | GDP-mannose 4,6-dehydratase |
| *wcaJ/H* | 6.2 | 0.0001 | Undecaprenyl-phosphate glucose phosphotransferase |
| *wcaB* | 4.9 | 0.0008 | Putative colanic acid biosynthesis acetyltransferase |
| *wcaC* | 4.8 | 0.0025 | Glycosyltransferase family 4 protein |
| *wcaD* | 5.0 | 0.0013 | Glycosyltransferase family 4 protein |
| *wcaE* | 5.0 | 0.0045 | Glycosyltransferase family 4 protein |
| *wzc* | 5.9 | 0.0008 | Protein-tyrosine kinase |
| *wzb* | 6.0 | 0.0007 | Tyrosine phosphatase |
| *wza* | 6.0 | 0.0012 | Putative polysaccharide export prote |
| *wzi* | 5.8 | 0.0025 | capsule assembly Wzi family protein |
| *galF* | 5.8 | 0.0005 | GalU regulator GalF |

**References**

[1] Q M, Z Z, Y L, J W, Z P, H Y, et al. Keeping alert to the hypervirulent K1, K2, K3, K5, K54 and K57 strains of Klebsiella pneumoniae within dairy production process. Microbes and Infection 2023;25. https://doi.org/10.1016/j.micinf.2023.105106.

[2] Datsenko KA, Wanner BL. One-step inactivation of chromosomal genes in Escherichia coli K-12 using PCR products. Proceedings of the National Academy of Sciences 2000;97:6640–5. https://doi.org/10.1073/pnas.120163297.
